# Heart patient health monitoring system using invasive and non-invasive measurement

**DOI:** 10.1038/s41598-024-60500-0

**Published:** 2024-04-26

**Authors:** Qurat-ul-Ain Mastoi, Ali Alqahtani, Sultan Almakdi, Adel Sulaiman, Adel Rajab, Asadullah Shaikh, Samar M. Alqhtani

**Affiliations:** 1 School of Computer Science and Creative Technologies, University of the West of England, Bristol, BS16QY UK; 2 Department of Networks and Communications Engineering, College of Computer Science and Information Systems, Najran University, 61441 Najran, Najran Saudi Arabia; 3 Department of Computer Science, College of Computer Science and Information Systems, Najran University, 61441 Najran, Saudi Arabia; 4 Department of Information Systems, College of Computer Science and Information Systems, Najran University, 61441 Najran, Saudi Arabia

**Keywords:** Health services, Cardiology, Diseases, Medical research

## Abstract

The abnormal heart conduction, known as arrhythmia, can contribute to cardiac diseases that carry the risk of fatal consequences. Healthcare professionals typically use electrocardiogram (ECG) signals and certain preliminary tests to identify abnormal patterns in a patient’s cardiac activity. To assess the overall cardiac health condition, cardiac specialists monitor these activities separately. This procedure may be arduous and time-intensive, potentially impacting the patient’s well-being. This study automates and introduces a novel solution for predicting the cardiac health conditions, specifically identifying cardiac morbidity and arrhythmia in patients by using invasive and non-invasive measurements. The experimental analyses conducted in medical studies entail extremely sensitive data and any partial or biased diagnoses in this field are deemed unacceptable. Therefore, this research aims to introduce a new concept of determining the uncertainty level of machine learning algorithms using information entropy. To assess the effectiveness of machine learning algorithms information entropy can be considered as a unique performance evaluator of the machine learning algorithm which is not selected previously any studies within the realm of bio-computational research. This experiment was conducted on arrhythmia and heart disease datasets collected from Massachusetts Institute of Technology-Berth Israel Hospital-arrhythmia (DB-1) and Cleveland Heart Disease (DB-2), respectively. Our framework consists of four significant steps: 1) Data acquisition, 2) Feature preprocessing approach, 3) Implementation of learning algorithms, and 4) Information Entropy. The results demonstrate the average performance in terms of accuracy achieved by the classification algorithms: Neural Network (NN) achieved 99.74%, K-Nearest Neighbor (KNN) 98.98%, Support Vector Machine (SVM) 99.37%, Random Forest (RF) 99.76 % and Naïve Bayes (NB) 98.66% respectively. We believe that this study paves the way for further research, offering a framework for identifying cardiac health conditions through machine learning techniques.

## Introduction

According to global statistics, around 735,000 Americans suffer from heart disease^1^. Moreover, the research conducted in China in 2011, named ’Report on Cardiovascular Diseases in China,’ reveals that about 230 million patients have CVD, with 3 million cases resulting in mortality yearly. This is estimated at around 41% of patients suffering from various heart disease issues^2^. In summary, heart disease is rapidly spreading across the globe, leading to a swift rise in mortality rates. The increasing incidence of heart disease can be attributed to several common factors, including obesity, issues related to cholesterol, drug use, and the neglect of critical heart conditions such as arrhythmia.

The long-term effect of arrhythmias could cause severe heart diseases, leading to death. Arrhythmia manifests in both life-threatening and non-life-threatening. It can be represented as irregular, slow, and fast heart rhythms^3^. However, to assess the arrhythmia, patients and doctors need to manually evaluate 24-hour ECG recording to determine the actual condition of the heart, which is a tedious process. Furthermore, using clinical data for diagnosing heart disease in patients is quite complicated and expensive. Therefore, researchers are seeking the attention of medical specialists to improve this field, aiming to reduce the expenses and time involved in diagnosing cardiac health conditions. Machine learning (ML) algorithms play a vital role in heart disease detection by leveraging the power of data analysis and pattern recognition. ML algorithms can continuously learn and adapt to new data and it is quite useful when patient is on continuous monitoring using wearable devices. ML help to detect subtle changes in heart rate, rhythm, and ECG patterns that might indicate the onset of a heart attack, arrhythmia, or other cardiac issues.Their ability to process large volumes of ECG signal data and identify the different variations in ECG helps healthcare providers in the early detection of patterns that may indicate an increased risk of heart disease.ML classifiers can provide more precise and accurate predictions compared to traditional methods. They can identify subtle patterns and correlations in data that might not be immediately apparent to human analysts, which saves patient lives, time, and healthcare costs. Several experiments have been performed on the automatic diagnosis of arrhythmia^4–7^ and heart disease classification using machine learning algorithms^8–13^. The automatic arrhythmia detection procedure includes signal processing, feature extraction, and implementation of learning algorithms for classification^14^. In contrast, the automatic heart disease detection procedure includes feature selection and classification^15^. Furthermore, the authors examined that the ECG signal is the core process or primary way to identify heart abnormality^14^. Therefore, the researcher used different techniques to assess and extract the most prominent clinical markers from the raw samples of ECG^16,17^ such as time-frequency analysis, higher-order cumulants, statistical analysis^18–20^, higher-order spectra^18^, spectral^21^.

The authors proposed system in study^22^ where they employ a fusion of three distinct sets of features: RR intervals, signal morphology, and higher-order statistics. The validation of this method utilized the MIT-BIH database following the inter-patient paradigm. Moreover, the system’s resilience to segmentation errors was assessed by introducing jitter to the R-wave positions extracted from the MIT-BIH database. Additionally, the robustness of each feature group against segmentation errors was individually tested.

Balamurugan^23^ introduced a system designed to rapidly detect abnormalities. The dataset, consisting of 75 attributes and 303 instances, was sourced from the UCI repository. The data underwent preprocessing and normalization to facilitate the selection of pertinent features. Utilizing image classification techniques, features were extracted from medical images. These extracted features were then subjected to clustering using the adaptive Harris hawk optimization (AHHO) approach. Subsequently, a deep genetic algorithm was employed for further classification. The proposed system demonstrated an accuracy of 97.3%, with its performance evaluated on the MATLAB/Simulink platform. Notably, the precision, sensitivity, and specificity metrics for the proposed method were recorded at 95.6%, 93.8%, and 98.6%, respectively. Nan et al^24^ employed a variety of classifiers for heart disease prediction. They utilized the Cleveland dataset sourced from the UCI repository, which comprised 270 records with 76 attributes. Notably, this study focused on utilizing only 13 attributes from the dataset. The prediction models employed included Support Vector Machine (SVM), Artificial Neural Network (ANN), and k-Nearest Neighbor (KNN). The SVM classifier achieved a classification accuracy of 85.18%. As for KNN, the accuracy steadily increased with an increasing value of k until reaching 80.74% at k=10. On the other hand, the ANN classifier yielded an accuracy of 73.33%.The majority of authors have relied on the UCI repository for heart disease detection. In our study, we explored two datasets to thoroughly examine the heart’s condition.The most important part of heart disease detection using UCI repository dataset is feature preprocessing. In the literature^9^, authors proposed a hybrid evolutionary technique for optimal features subset^25^, swarm intelligence-based artificial bee colony(ABC) feature selection^26–28^, genetic algorithm for heart disease features selection^29^. These feature extraction and feature selection methods are further combined with different state-of-the-art learning algorithms such as the authors used SVM+NN classifiers to predict arrhythmia^31^, SVM with the radial basis for multi-disease prediction^32^, KNN proposed arrhythmia detection^33,34^, random forest performs overwhelmingly in the prediction of heart disease^35^, Levenberg-Marquardt -NN^36^ and artificial neural network^15,37,38^. Researchers have done many extensive experiments in the past and demonstrated various achievements in predicting heart arrhythmia and heart diseases.

According to the author’s information, there is a lack of a framework that can utilize a feature preprocessing approach using two distinct datasets: MIT-BIH-arrhythmia **(DB-1)** and Cleveland heart disease **(DB-2)** for the complete analysis of cardiac health conditions is lacking. Secondly, to prevent biased diagnoses, this study introduces a novel approach to determining the certainty level of machine learning models using information entropy. The term information entropy describes the information of uncertainty in the events^39,40^, which was created by mathematician Claude Shannon^39^. This concept is innovative in determining the effectiveness of machine learning algorithms and has not been previously explored in computational biology studies. The proposed study conducted extensive experiments to minimize biased diagnoses of cardiac health conditions. We anticipate that this research will pave the way for a new direction in machine learning by introducing the information entropy mechanism to calculate the uncertainty level of conventional learning algorithms applied in the current study.

The highlighted aim of our proposed framework:

1. Extensive feature engineering was employed to extract six key features from the ECG waveforms.
2. To evaluate the efficiency of the proposed feature extraction approach in terms of accuracy, sensitivity, and detection error rate.
3. Proposed algorithm to analyze the behaviour of beats.
4. To predict cardiac health conditions by analyzing the behaviour of the beats in terms of abnormal arrhythmia beats and heart disease using machine learning algorithms.
5. To evaluate the performance of learning algorithms by using the information theory concept (information entropy).

Implementation of this method will significantly assist medical specialists in identifying cardiac health using different datasets. Our proposed methodology demonstrates exceptional performance in diagnosing cardiac health conditions in terms of arrhythmia and heart disease. The remainder of the paper is structured as follows: The author explained the materials and methods used in this experiment step by step after the introduction section. The next section discusses the experimental settings. After that, we describe calculation of information entropy. In the end, the authors define the results, conclusion, and future work.

## Materials and methods

### The proposed methdology

**Figure 1:**
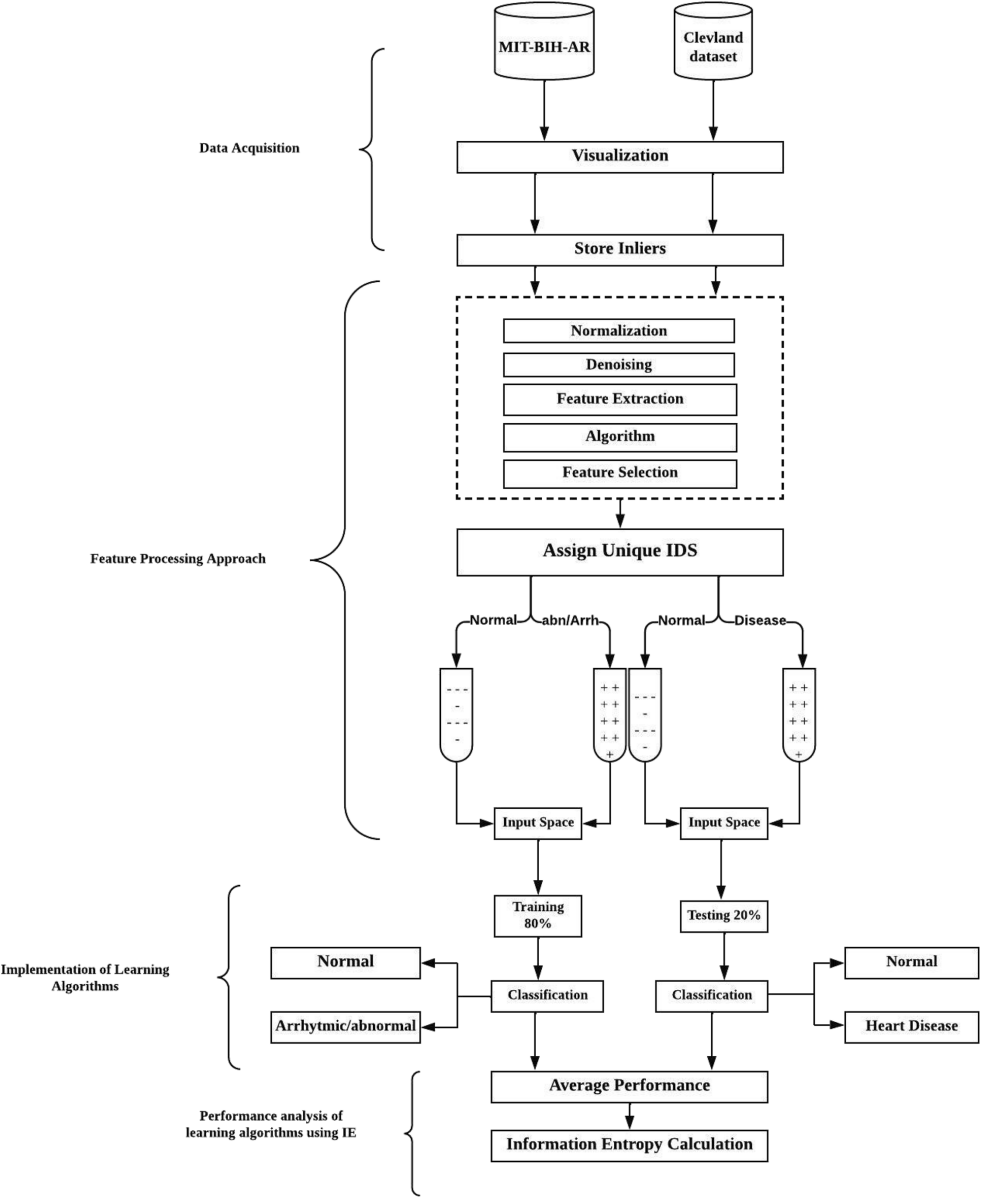
The overall proposed framework of the feature preprocessing Approach.

The main goal of this research is to preprocess the datasets (DB-1) and (DB-2) and extract/choose relevant features that aid in diagnosing cardiac health conditions related to arrhythmia, abnormal beats, and heart disease. Furthermore, this research includes a novel experiment that analyzes the average uncertainty level of the classifier using Information Entropy; applying this concept is absolutely a unique factor and not previously been explored in cardiac abnormality detection. The workflows of the overall proposed framework are represented in Fig. 1. Our proposed framework comprises four main steps, two of which involve the preprocessing of datasets (DB-1) and (DB-2). The last two step involve in prediction of cardiac health conditions and performance analysis of the learning algorithms. This section outlines the experimental process in the following steps:

### Data acquisition

This step constitutes a significant part of the study. We gathered datasets from public sources and implemented a straightforward preprocessing technique based on the advice from these sources. The details of the dataset are explained below:

*(DB-1):* The MIT-BIH Arrhythmia Dataset and AAMI Standards.

The dataset consisted of 48 half-hour records of two leads (MLII), and V1 were obtained from 47 subjects^44^. Over a 10mV range, the signals were captured at a sampling frequency of 360Hz and a resolution of 11 bits. The dataset was divided into groups’ normal and arrhythmia/abnormal, 25 and 23 ECG segments, respectively. Furthermore, this study followed the AAMI standard for arrhythmia classification. According to the AAMI (Association for the Advancement of Medical Instrumentation), the MIT-BIH arrhythmia dataset has four recordings (102,104,107, and 217) containing paced beats because the signal did not retain sufficient signal quality for automatic prediction. Therefore, the study used the rest of the 44 recordings (Lead II) for our experiments.

*(DB-2):* UCI Repository for Machine Learning Dataset. The Cleveland Clinic Foundation provided the database for heart disease classification^42^. This database consisted of 76 parameters, out of which only 14 parameters with 303 instances were presented for experiments (see Table 1). In the acquisition section, we observed that 33 instances have missing values. Due to that reason, only 270 instances were taken for this experiment.

**Table 1:** Clinical attributes of DB-2.

Features	Description	Values
Age	Age	29–77
Sex	Sex	1 = male,0=female
Cp	Chest pain type	1 = typical angina, 2 = atypical angina, 3=non-angina and 4= asymptomatic pain
Trestbps	Resting blood pressure on admission	(94,200)
Chol	Serum cholesterol(mg/dl)	(126,564)
Fbs	Fasting blood sugar (>120 mg/dl)	1 = true and 0 = false
Restecg	Resting ECG outcome	(0,2)
Thalach	Maximum heart rate achieved	(71,202)
Exang	Exercise induced angina	1 = yes and 0 = no
Oldpeak	ST depression induced by exercise related to rest.	(0.00 ,62.00)
Slope	The slope of the peak exercise ST segment	1 = upsloping, 2 = flat and 3 = downsloping
Ca	Number of fluoroscopy-colored vessels	(0,3)
Thal	Reversible defect and class	3 = normal and 6 = fixed defect

### Feature prepocessing approach

The literature^44–46^ emphasizes that the preprocessing stage is the fundamental prerequisite step of every classification technique because an unpreprocessed feature set directly affects their final analysis. Thus, medical diagnosis directly impacts human lives; therefore, ensuring unbiased feature sets during diagnosis is crucial. Our study emphasizes the significance of properly preprocessing cardiac-related features from (DB-1) and (DB-2) in diagnosing cardiac health conditions. ECG signals serve as the primary source for understanding of cardiac health conditions. Therefore, the author’s main focus is to preprocess ECG signals accurately. The feature preprocessing approach involves the following steps:

#### Normalization

Normalization is the process of reducing the DC offset and eliminating amplitude variance for each ECG signal. According to Mark et al., it is necessary to normalize the ECG signal^41^ due to a potential source of clicks and distortion. The equations of the normalization process of raw ECG signal are determined as follows:

1 $$\begin{aligned} \bar{x_j}(i) = 2. \left( \frac{x_j (i) - min (x_j)}{max (x_j) - min (x_j)}\right) -1 \end{aligned}$$

where *i* represents the index of consecutive ECG signal samples, *j* represents the index of consecutive ECG signals, min$x_j$ represents the minimum signal amplitude value, and max $x_j$ represents the maximum signal amplitude value.

#### Filtering

The contaminated ECG signals were the major problem in the bio-electrical records, as discussed in^47,48^. ECG signals contain a variety of distortions, such as low-frequency noises, baseline drifting^49,50^, and high-frequency noises like the power line interface^51,52^. The power-line interface comprises a 50Hz pickup with an amplitude of 50% from peak to peak. However, baseline wandering is mostly induced by the patient’s breathing or movement, which creates hurdles in recording ECG peaks. Due to these different artefacts, the raw ECG signals cannot be used directly to seek the information of interest because it may lead to the wrong diagnosis of cardiac health conditions. To eliminate these types of noises, this study used a simple finite impulse response(FIR) and notch filters to remove the contamination part from the ECG signal as proposed by^53–58^ for low-frequency and high-frequency noise, respectively.

#### Feature extraction

**Figure 2:**
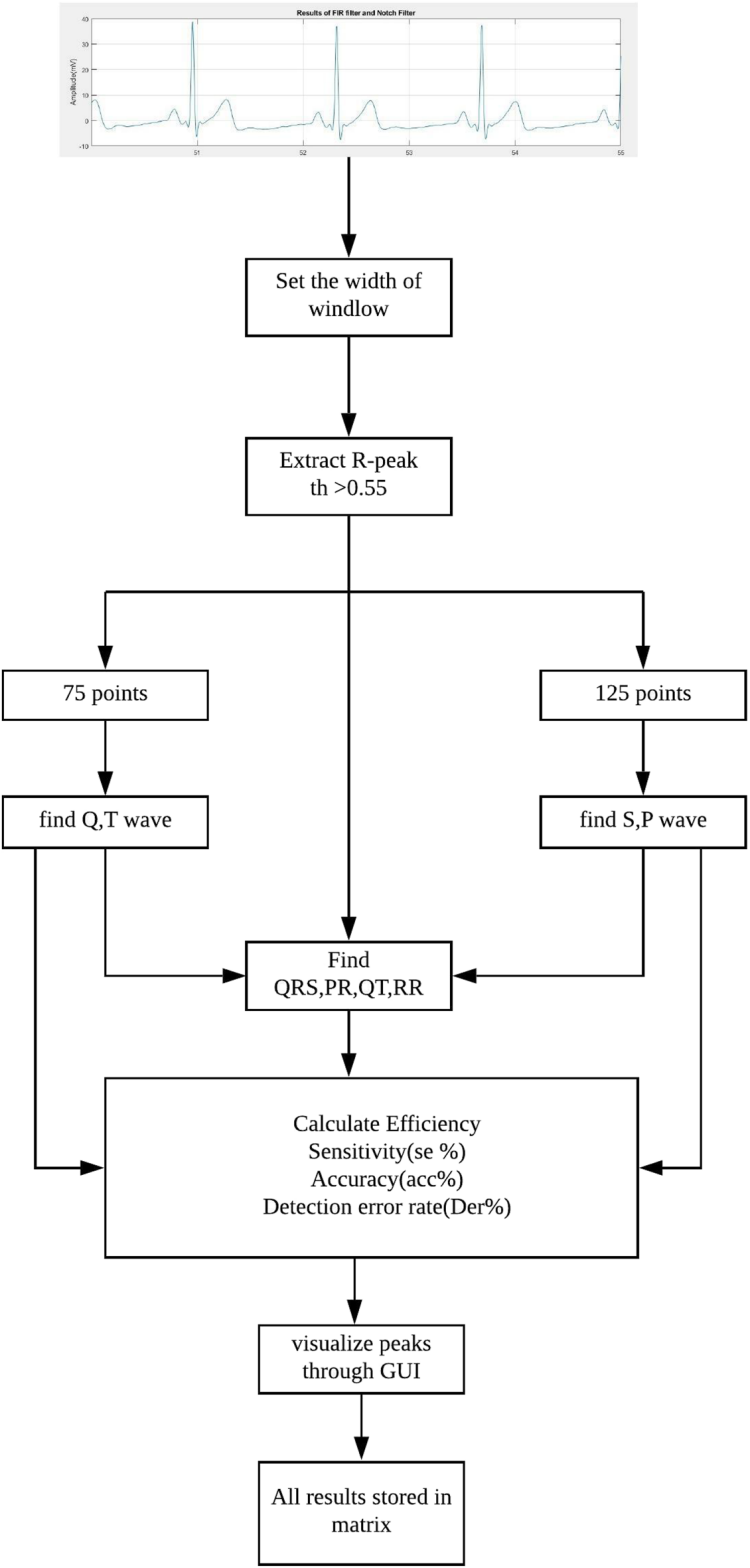
The overall process of feature extraction.

This section is dedicated to extracting the essential clinical markers from ECG signals to analyse normal, abnormal, and arrhythmic beats. This phase has been executed based on the recommendations of clinical experts. The overall process of feature extraction is explained in Fig. 2. Initially, the author established the window width with a constant sampling frequency of 360Hz, a parameter already set or provided by https://physionet.org/^55^.

This stage of the feature extraction involves modifying the Pan and Tompkins algorithm to accurately detect the R-peak value. The reason for modifying the conventional technique is to identify the negative amplitude parameter of the R-peak from ECG signals. Although the Pan and Tompkins algorithm is a common algorithm used in many existing studies, the conventional Pan and Tompkins algorithm did not accurately return the negative polarity values of the QRS complex. In the modification part of the Pan and Tompkins algorithm we have introduced Local Maxima and Minima Difference (LMMD) through an adaptation of discrete Morse theory^36^ which enable algorithm to extract the distance between the positive and negative peaks on the QRS complex.Adopting the modified Pan and Tompkins algorithm in this study lies in its capacity to accommodate pronounced variations in ECG signals and ascertain precise threshold values for R-peak extraction. To delve further into the analysis of ECG cycle features, 200 samples are chosen around the R-peak, comprising 75 points from the left side and the remainder from the right side of the R-peak. This main feature aids in the automatic detection of QRS by extracting the minimum values from both the left and right sides.

The signal is divided based on the window width, and the high-and lowest frequency component within that particular segment of the ECG signal is identified by establishing a threshold value. A vector matrix **lrp** is created to store the R-peak values with the indexes and reference points for calculating the related peaks of ECG signals.

**Figure 3:**
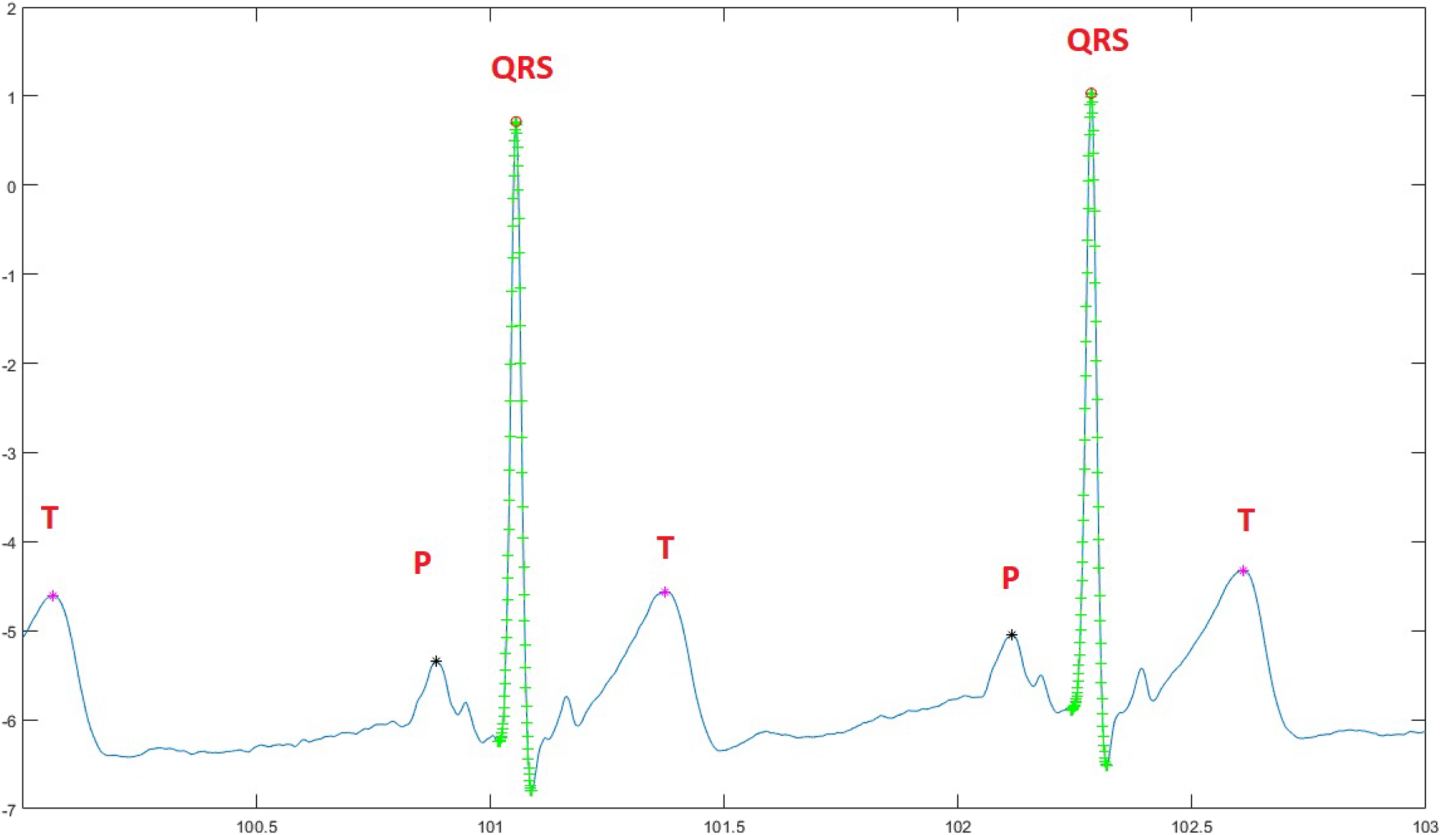
Detection of main Peaks.

Moreover, the identification of T and P peaks involved the use of 200 points in a similar manner. Figure 3 illustrates the extraction of all pertinent features from ECG signals. Additionally, this research determines the distance between the positive and negative peaks on the QRS complex by introducing the Local Maxima and Minima Difference (LMMD) through an adaptation of discrete Morse theory^36^.This computational approach computes amplitude differences between high and low amplitudes by eliminating the smallest difference in each pass-over. This technique is applied to cancel the smallest amplitude until the desired threshold is achieved in the sequence. However, for the detection of the negative peaks in the QRS complex, the minimum value should exhibit the most significant amplitude difference ratio compared to other prominent peaks. After applying the appropriate threshold value using ADMT, the remaining peaks were categorized as S-waves and R-waves. To establish the threshold value for the largest amplitude, the authors employed the unsupervised k-means clustering technique. Two clusters were defined, and the threshold values were specified as Eq. (2).

2 $$\begin{aligned} th = \frac{max (w_0+min(w_1))}{2} \end{aligned}$$

where $w_0$ is the cluster that contains the smallest amplitude value, and the largest amplitude value was contained by $w_1$. This technique aids in detecting the complete QRS area from the ECG signal. After the feature extraction process, all the extracted features were stored in a matrix denoted as “fpi” to classify normal, abnormal, and arrhythmia-beat behaviours. The extracted attributes include the time duration of the R-R interval, QRS, QT interval, T-wave, PR interval, and P-wave.

*Proposed algorithm for predicting the behaviour of beat (normal, abnormal, and arrhythmic)* The major role of Algorithm 1 is to classify the different behaviours of the beats for instance, normal, abnormal and arrhythmic. Moreover, to improve and verify the accuracy of this algorithm, we considered expert suggestions and clinical information to verify the results. The normal ranges of the waves and peaks are defined as follows:

1. The distance between two R consecutive beats should not be greater than 1.2 seconds. If the distance ratio between two subsequent beats increases, it might have a chance to get an arrhythmic beat^57,59,60^.
2. The normal QRS duration value is between 0.12 s and 0.20 s. Suppose the duration of this complex increases, and the irregular R-R interval is also present. In that case, it may get premature ventricular contraction beats(PVC) because this type of arrhythmia has much higher amplitudes^61^.
3. The duration from the Q-wave to T-wave has to be less than 0.44 s^62^.
4. The normal duration between P-wave to R-wave has to be situated between 0.12 s and 0.20 s^63^.

**Algorithm 1:**
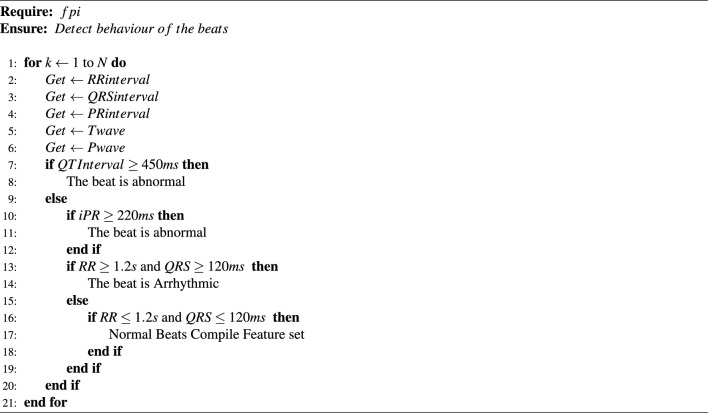
Steps for predicting the behaviour of beats (normal, abnormal and arrhythmic beats

#### Stepwise regression for feature selection

The stepwise regression technique is the intuitive approach for including and excluding attributes from the dataset based on a regression analysis of their statistical data^63^. The primary procedure of stepwise regression is to analyze the data based on the regression analysis. The major benefit of this strategy is that it is a mixture of the forward and backward selection methods. Therefore, this method tests the variable at each step for adding or removing using forward for selection and backward for elimination, respectively^64^.The stepwise regression method can manage large amounts of potential predictor variables and fine-tune the model to select the best predictor variables. The most important factor to consider in this method for parameter selection is that it is faster than other automatic model-selection methods. This method used only to preprocess DB-2 , as this dataset is well-ordered,therefore we only reduce the dimension of the features .To implement this method we followed below procedure.

1. Initially, we begin by comparing the explanatory power of successively bigger and smaller models. To test models with and without a potential term, the p-value of an F-statistic is generated at each stage.
2. At every step of the model, the algorithm calculates a p-value to test the model to get the potential term added to the model and this process is repeated else move to step 3.
3. In the third phase of the algorithm, it checks whether any possible term has a p-value larger than the exit tolerance, eliminates the one with the highest p-value, and repeats step 2 if necessary; otherwise, the process terminates^65^.

#### Assigning unique identifiers

**Figure 4:**
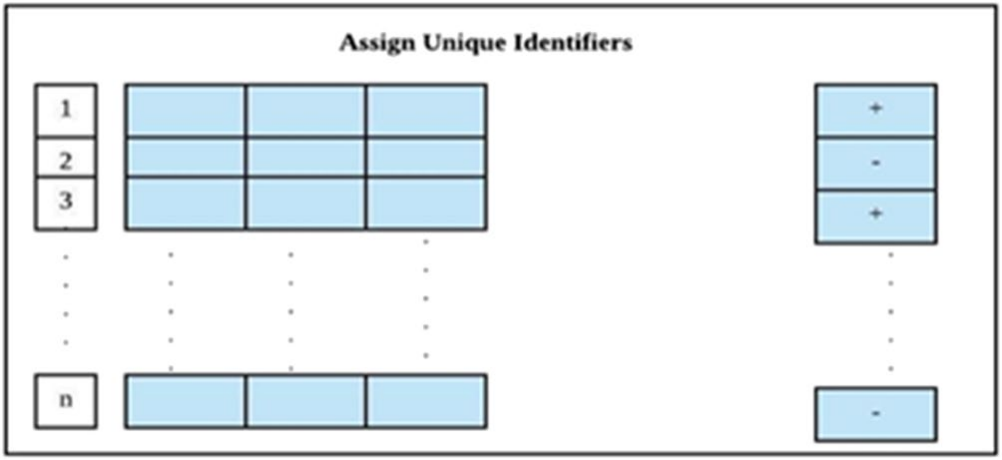
Assigning unique identifiers to all attributes.

At this stage of the study, we assign a unique identifier to each attribute in both datasets (DB-1 and DB-2) (refer to Fig. 4). Subsequently, a distinct input space is generated to validate the outcomes derived from learning algorithms.

### Implementation of learning algorithms

In this section ,we select five different learning algorithms for instance k-nearest neighbour, neural network, support vector machine, random forest, and Naive Bayes .To validate the result ,we divided datasets in two parts substantial amount of data, around 80% for training and 20% for testing the learning algorithms. Moreover, this study chooses to utilize the k-fold cross-validation technique, setting k to 10, to properly evaluate the performance of classifiers.

#### K-nearest neighbor (KNN)

The KNN^66^ is one of the perspectives and non-parametric classification method based on the minimum distance classifier, or it can also be defined as KNN classifying objects based on the closest training values in the feature space^64^. This algorithm is widely used for arrhythmia and heart disease classification^68–71^. This algorithm’s learning procedure involves comparing the training dataset’s input feature vector with the unlabeled dataset for testing. We classify the normal and abnormal data by categorizing query points and their distance to points in a training dataset. However, in the KNN algorithm, the training phase is very fast, but the testing phase is too costly in terms of time and memory^72^. The k-nearest neighbors (k-NN) algorithm is based on the equation:

3 $$\begin{aligned} {\hat{y}} = \text {mode}\left( \{y_i\}_{i \in \text {NN}(x)}\right) \end{aligned}$$

where:

- ${\hat{y}}$ is the predicted label for the input *x*.
- $\text {mode}$ is the function that returns the most common label among the *k* nearest neighbors.
- $y_i$ is the label of the *i*-th neighbor.
- $\text {NN}(x)$ is the set of indices corresponding to the *k* nearest neighbors of *x*.

In this equation, the predicted label ${\hat{y}}$ for a new input *x* is determined by finding the *k* nearest neighbors of *x* from the training dataset, and then selecting the most common label among these *k* neighbors.

#### Neural network

This algorithm comprises highly interconnected processing elements and layers that process information through their dynamic state to an external state of the algorithm. The neural network (NN) recognizes underlying relationships within the dataset similar to how the human brain works. In our study, we utilized this algorithm with five layers, specifically two layers of input and output, and the remaining layers are hidden, containing a certain number of interconnected nodes. We employed the tanh activation function with seven neurons to classify the cardiac health condition from the dataset. The input space vector is fed through an input layer connected to other operational hidden layers. After processing, the output layer receives the response vector from the hidden layer. The neural network can adapt to variations in input, allowing the network to generate the best possible result without redesigning the output criteria^73,74^. The equation for the output ${\textbf{y}}$ of a neural network layer is:

4 $$\begin{aligned} {\textbf{y}} = \sigma (\textbf{Wx} + {\textbf{b}}) \end{aligned}$$

where:

- ${\textbf{x}}$ is the input to the layer (a vector of size *n*).
- ${\textbf{W}}$ is the weight matrix of the layer (an $m \times n$ matrix, where *m* is the number of neurons in the layer).
- ${\textbf{b}}$ is the bias vector (a vector of size *m*).
- $\sigma$ is the activation function applied element-wise to the result of $\textbf{Wx} + {\textbf{b}}$.

In this equation, the predicted label ${\hat{y}}$ for a new input *x* is determined by finding the *k* nearest neighbors of *x* from the training dataset, and then selecting the most common label among these *k* neighbors.

#### Support vector machine (SVM)

The support vector machine is a supervised learning algorithm, formally defined by a separating hyperplane. SVM analyzes data through classification and regression analysis and is widely utilized in cardiac studies for classification problems^75^. Therefore, we also employed SVM to diagnose the cardiac health condition from the input feature vector. SVM efficiently performs non-linear classification using kernel methods to implicitly map feature vectors into high-dimensional feature spaces^76–78^. The learning method of this algorithm employed the radial basis function kernel method^79^, a real-valued function whose value depends solely on the distance from the main origin^80^. SVM classifiers construct a hyperplane with n dimensions, where n indicates the number of attributes in the input feature vector. Based on our training and testing criterion, the hyperplane divides the input feature vector into train and test, labeled and unlabeled, respectively. The equation for a linear Support Vector Machine (SVM) is given by:

5 $$\begin{aligned} y({\textbf{x}}) = {\textbf{w}}^T {\textbf{x}} + b \end{aligned}$$

where:

- $y({\textbf{x}})$ is the predicted output for input ${\textbf{x}}$.
- ${\textbf{w}}$ is the weight vector.
- ${\textbf{x}}$ is the input vector.
- *b* is the bias term.

In this equation, the decision boundary is defined by the hyperplane ${\textbf{w}}^T {\textbf{x}} + b = 0$. The sign of $y({\textbf{x}})$ determines the predicted class label, where $y({\textbf{x}}) > 0$ corresponds to one class and $y({\textbf{x}}) < 0$ corresponds to the other class.

#### Random forest

This algorithm was initially developed and introduced by Breiman^81^. In our study, we applied this supervised learning algorithm to our feature vector to distinguish between normal and abnormal classes. Random Forest (RF) generates a random vector using our feature set, where all values of the random vector are independent. Throughout this procedure, RF constructs a set of tree-structured classifiers for training and testing labeled and unlabeled datasets. The Random Forest algorithm combines predictions from multiple decision trees. The prediction for a Random Forest model can be represented as:

6 $$\begin{aligned} {\hat{y}} = \frac{1}{N} \sum _{i=1}^{N} f_i({\textbf{x}}) \end{aligned}$$

where:

- ${\hat{y}}$ is the predicted output for input ${\textbf{x}}$.
- *N* is the number of trees in the Random Forest.
- $f_i({\textbf{x}})$ is the prediction of the *i*-th decision tree for input ${\textbf{x}}$.

In this equation, the Random Forest model aggregates the predictions of individual decision trees to make the final prediction ${\hat{y}}$ for the input ${\textbf{x}}$.

#### Naïve Bayes

Naïve Bayes is a sophisticated classification algorithm based on the Bayesian theorem, belonging to the family of simple probabilistic classifiers^82^. This technique is easy to build, simple, and effective for large feature vectors. In our study, we utilized this classifier to assess the algorithm’s performance with our prepared dataset. Despite its apparent simplicity, the algorithm can often deliver outstanding performance in classification using our feature set. The Naive Bayes classifier predicts the probability of a class $C_k$ given an input feature vector ${\textbf{x}}$ using Bayes’ theorem:

7 $$\begin{aligned} P(C_k | {\textbf{x}}) = \frac{P({\textbf{x}} | C_k) P(C_k)}{P({\textbf{x}})} \end{aligned}$$

where:

- $P(C_k | {\textbf{x}})$ is the probability of class $C_k$ given input ${\textbf{x}}$.
- $P({\textbf{x}} | C_k)$ is the likelihood of observing ${\textbf{x}}$ given class $C_k$.
- $P(C_k)$ is the prior probability of class $C_k$.
- $P({\textbf{x}})$ is the probability of observing ${\textbf{x}}$.

The Naive Bayes assumption assumes that the features are conditionally independent given the class label. This simplifies the likelihood term:

8 $$\begin{aligned} P({\textbf{x}} | C_k) = \prod _{i=1}^{n} P(x_i | C_k) \end{aligned}$$

where:

- $x_i$ is the *i*-th feature of ${\textbf{x}}$.

The class with the highest probability $P(C_k | {\textbf{x}})$ is predicted by the Naive Bayes classifier.

## Experimental settings

In the experiments, we introduced a method for preprocessing two distinct datasets (DB-1 and DB-2) to diagnose cardiac health conditions related to abnormal arrhythmic beats and heart disease. Our study placed a specific emphasis on assessing classifier performance. Information entropy was utilized to gauge the level of uncertainty, employing five different learning algorithms. All preprocessing of the datasets was carried out using MATLAB 2016b. Additionally, we utilized an orange data mining Python-based tool for assigning unique IDs and training the learning algorithms with our preprocessed feature set. The experiments were conducted on a 64-bit Windows 10 system with an Intel(R) Core(TM) i7-3770 CPU running at 3.40GHz and 6GB of RAM.

### Performance metrics

Our study conducted a comprehensive analysis to assess the performance of our proposed method. The study employed ten performance metrics, including the area under the curve (AUC), classification accuracy (ACC), precision, recall, F1 score, Mathews correlation coefficient (MCC), false positive rate (FPR), sensitivity (Se), specificity (Sp), and G-mean The definitions of these metrics are provided below:

1. The area under the curve is defined as (3)
  9 $$\begin{aligned} { AUC= \frac{1}{2} \left( \frac{TP}{TP+FN} + \frac{TN}{TN+FP}\right) } \end{aligned}$$
2. The classification accuracy is the most important metric for evaluating the performance of the classifier, is defined as (4)
  10 $$\begin{aligned} ACC = \frac{TN+TP}{TP+FP+FN+TN} \end{aligned}$$
3. The precision or positive predictively is defined as (5)
  11 $$\begin{aligned} prec = \frac{TP}{TP+FP} \end{aligned}$$
4. Sensitivity is defined as (6)
  12 $$\begin{aligned} Se = \frac{TP}{TP+FN} \end{aligned}$$
5. The F1-Score is defined as (7)
  13 $$\begin{aligned} F1 = 2. \frac{precision.Recall}{precision+Recall} \end{aligned}$$
6. The Mathews correlation coefficient is defined as (8)
  14 $$\begin{aligned} \begin{aligned} MCC = \frac{TP*TN-FP*FN}{\sqrt{(TP+FP)(TP+FN)(TN+FP)(TN+FN)}} \end{aligned} \end{aligned}$$
7. The false-positive rate is defined as (9)
  15 $$\begin{aligned} FPR = \frac{FP}{FP+TN} \end{aligned}$$
8. The specificity is defined as (10)
  16 $$\begin{aligned} SP = \frac{TN}{TN+FP} \end{aligned}$$
9. The G-mean is defined as (11)
  17 $$\begin{aligned} G-mean = \sqrt{precision*ReXcall} \end{aligned}$$
10. The Detection error rate is defined as (12)
  18 $$\begin{aligned} DER = \frac{FP+FN}{TP+FP+FN+TN} \end{aligned}$$

where,

True negative (TN) samples of normal records which are correctly classified as normal; True positive (TP) samples of abnormal records which are correctly classified as abnormal; False-positive (FP) samples of normal records which are classified as abnormal records; False Negative (FN) samples of abnormal records are classified as normal.

## Information entropy

Information entropy is a fundamental concept in Information theory that characterizes the amount of information present in an event. The concept revolves around calculating the level of uncertainty associated with the value of an event derived from a random variable or obtained from the outcomes of a random process^83,84^.

In this study, we employed the Information Entropy method on the outputs of the selected classifier to assess the impurity of classifiers. We consider the diagnosis of cardiac health conditions a sensitive area of study, emphasizing the need for a thorough evaluation of classifier performance. This aspect can be viewed as a distinctive contribution to our research, as previous studies have not extensively focused on evaluating classifier performance in medical contexts. Nonetheless, we applied Information Entropy to the outputs of five distinct learning algorithms to assess the performance of the most suitable algorithm for our preprocessed feature set.Logarithm base is set with 2, and $p_i$ is the information entropy’s probability function, which is equal to $\frac{1}{2}$. The following equation defines the information entropy measurement for learning algorithms.

19 $$\begin{aligned} H(x) = -\sum _{i=1}^{n}p_i log_2 p_i \end{aligned}$$

20 $$\begin{aligned} H(x) = \sum -P_{perf} log_2 P_{perf} - P_{diff} log_2 P_{diff} \end{aligned}$$

where $P_{perf}$ and $P_{diff}$ are defined as the classifier’s performance and difference ratio concerning 100% of classifier, respectively.

## Results and discussions

To showcase the effectiveness of our feature extraction process based on (DB-1), we utilized sensitivity, accuracy, and detection error rate (DER) to evaluate the efficiency of our extracted features. Table 2 illustrates the effectiveness of the feature extraction technique. Following the feature preprocessing approach using (DB-1), our study then proposed a simple model, guided by medical advice, to classify normal, abnormal, and arrhythmic beats from the stored record of features. The primary purpose of constructing this model is to provide input for classifiers by discerning positive and negative attributes of feature sets using (DB-1). However, this method is efficient enough to separate all normal, abnormal, and arrhythmic beat values.

The model’s performance is assessed using the DB-1 dataset, a benchmark dataset for electrocardiogram (ECG) analysis. the same performance metrics were employed as those used to evaluate the performance of the feature extraction algorithm Table 3. The model’s ability to accurately detect and classify heartbeats is crucial for its efficacy in real-world applications. Moreover, the model’s proficiency in determining the total number of beats, denoted as NCB, serves as a fundamental metric of its effectiveness. NCB represents the comprehensive count of all detected heartbeats within the ECG signals. A higher NCB indicates the model’s capability to accurately identify individual heartbeats, essential for tasks such as heart rate monitoring and arrhythmia detection.Moreover, the model’s prowess in analyzing missing beats (NMB) provides valuable insights into its robustness and reliability. NMB signifies the model’s capacity to detect gaps or irregularities in the ECG signals, indicating potential missed heartbeats or abnormal rhythms. A lower NMB suggests that the model can effectively perform well in identifying and analyzing missing beats, highlighting its accuracy and reliability in ECG signal analysis.The achieved results highlight the comprehensive performance of our proposed model, boasting a sensitivity (se) of approximately 99.98%, an accuracy (acc) of 99.97%, and an impressively low detection error rate of 0.025.

**Table 2:** Overall performance of feature extraction using DB-1.

Feature	SE%	ACC%	DER%
R-R interval	99.99	99.98	0.01
QRS complex	99.99	99.98	0.01
QT interval	99.99	99.99	0.005
P-R interval	99.99	99.99	0.005
P-wave	99.99	99.99	0.004
T-wave	99.99	99.99	0.004

**Table 3:** Results of the proposed algorithm for identification of beats.

Records	Beats	CB	NMB	SE%	ACC%	DER%
100	2272	2272	0	100	100	0
101	1862	1862	0	100	100	0
103	2084	2084	0	100	100	0
105	2570	2570	0	100	100	0
106	2026	2024	2	99.90	99.80	0.19
108	1762	1762	0	100	100	0
109	2532	2532	0	100	100	0
111	2122	2122	0	100	100	0
112	2538	2538	0	100	100	0
113	1794	1794	0	100	100	0
114	1878	1878	0	100	100	0
115	1952	1952	0	100	100	0
116	2412	2409	3	99.91	99.83	0.16
117	1534	1534	0	100	100	0
118	2276	2276	0	100	100	0
119	1986	1984	2	99.89	99.79	0.20
121	1862	1862	0	100	100	0
122	2476	2476	0	100	100	0
123	1516	1516	0	100	100	0
124	1618	1618	0	100	100	0
200	2600	2598	2	99.92	99.84	0.15
201	1962	1962	0	100	100	0
202	2136	2136	0	100	100	0
203	2978	2978	0	100	100	0
205	2656	2656	0	100	100	0
207	1860	1858	2	99.89	99.89	0.10
208	2954	2954	0	100	100	0
209	3004	3004	0	100	100	0
210	2650	2650	0	100	100	0
212	2748	2748	0	100	100	0
213	3250	3249	1	99.93	99.93	0.06
214	2262	2262	0	100	100	0
215	3362	3362	0	100	100	0
219	2154	2154	0	100	100	0
220	2046	2046	0	100	100	0
221	2426	2426	0	100	100	0
222	2482	2482	0	100	100	0
223	2604	2604	0	100	100	0
228	2052	2052	0	100	100	0
230	2256	2256	0	100	100	0
231	1570	1570	0	100	100	0
232	1780	1779	1	100	99.88	0.11
233	3078	3076	2	99.93	99.87	0.13
234	2752	2752	0	100	100	0
Avg/Total	100,694	100,679	15	99.98	99.97	0.025

We utilized a stepwise fit feature selection algorithm to preprocess the DB-2 clinical dataset, focusing solely on feature selection without the need for feature extraction. The analysis revealed that 7 attributes out of the total 14 exhibited notably improved efficiency. The findings of the stepwise fit algorithm are detailed in Table 4.

**Table 4:** Results of the stepwise fit method.

Selected attributes	*P*-value
Cp	0.0290
Thalach	0.0059
Exang	0.0293
Oldpeak	0.091
Slope	0.0166
Ca	0.0111
Thal	0.0368

### Performance of learning algorithms

In this study, we assess our preprocessed feature sets with five different learning algorithms to obtain the best classifier results. Our proposed method employs 80% of the data for training the classifiers and reserves 20% for testing. The rationale for using a substantial amount of data for training is to ensure that our proposed learning algorithm was not developed using contaminated data and that our training data did not yield biased results. Furthermore, a 10-fold cross-validation technique is utilized to validate the classifier’s performance.

We used five classifiers to accurately predict the cardiac health condition in terms of four classes: normal, abnormal beats, arrhythmia beats, and heart disease using the extracted and selected feature set. Table 5 displays the statistics of the MIT-BIH-arrhythmia dataset (DB-1), while Table 6 presents the results of the heart disease dataset (DB-2). Additionally, Tables 7 and 8 illustrate the average classifier performance values and different ratios of our average performance results. These results can help calculate the information of uncertainty. The terms defined in Tables 5, 6, 7, and 8 are area under the curve (AUC), classification accuracy (ACC), F1-score, precision (prec), Mathew’s correlation coefficient (MCC), false-positive rate (FPR), sensitivity (Se), specificity (Sp), and G-mean.

**Table 5:** Results of classifiers using (db-1) mit-bih arrhythmia dataset.

classifier	AUC	ACC	F1	Prec	MCC	FPR	SE	SP	G-mean
Nn	1.000	0.998	0.995	0.996	0.994	0.0009	0.994	0.999	0.995
Knn	0.998	0.994	0.984	0.987	0.98	0.0027	0.98	0.997	0.983
SVM	0.999	0.998	0.994	0.994	0.99	0.001	0.994	0.998	0.994
RF	1.000	0.999	0.997	0.998	0.99	0.002	0.996	0.999	0.997
Nb	0.997	0.980	0.945	0.930	0.933	0.07	0.961	0.983	0.945

**Table 6:** Results of classifiers using (db-2) heart disease dataset.

Classifier	AUC	ACC	F1	Prec	MCC	FPR	SE	SP	G-mean
Nn	0.874	0.806	0.806	0.806	0.611	0.193	0.804	0.806	0.649
Knn	0.713	0.690	0.690	0.691	0.381	0.339	0.720	0.660	0.690
SVM	0.798	0.731	0.730	0.737	0.468	0.333	0.799	0.666	0.733
RF	0.858	0.783	0.783	0.783	0.565	0.220	0.786	0.799	0.783
Nb	0.885	0.803	0.806	0.804	0.527	0.215	0.822	0.784	0.803

**Table 7:** Average classifiers performance.

Classifier	AUC	ACC	F1	Prec	MCC	FPR	SE	SP	G-mean
Nn	0.937	0.902	0.900	0.901	0.802	0.096	0.899	0.902	0.822
Knn	0.855	0.842	0.837	0.839	0.680	0.170	0.85	0.828	0.836
SVM	0.898	0.864	0.862	0.865	0.729	0.167	0.896	0.832	0.8635
RF	0.929	0.891	0.89	0.890	0.777	0.111	0.891	0.899	0.89
Nb	0.941	0.891	0.875	0.867	0.73	0.145	0.891	0.883	0.874

**Table 8:** Difference ratio of average classifiers performance.

Classifier	AUC	ACC	F1	Prec	MCC	FPR	SE	SP	G-mean
Nn	0.063	0.098	0.1	0.099	0.198	0.904	0.101	0.098	0.178
Knn	0.145	0.158	0.163	0.161	0.32	0.83	0.15	0.172	0.164
SVM	0.102	0.136	0.138	0.135	0.271	0.833	0.104	0.168	0.137
RF	0.071	0.109	0.11	0.11	0.223	0.889	0.109	0.101	0.11
Nb	0.059	0.109	0.125	0.133	0.27	0.855	0.109	0.167	0.126

The results from Tables 5, 6, 7, and 8 were utilized to calculate the uncertainty information of five different learning algorithms. Additionally, we used the average classifier performance results from Table 8 and Table 9 for information entropy calculation. Meanwhile, Table 10 presents the results of the calculated information entropy of the five different learning algorithms, using the same performance metrics discussed in Tables 5, 6, 7, 8.

**Table 9:** Information entropy results in bits.

Classifier	AUC	ACC	F1	Prec	MCC	FPR	SE	SP	G-mean
Nn	0.325	0.461	0.468	0.464	0.716	0.455	0.471	0.461	0.662
Knn	0.595	0.628	0.640	0.635	0.903	0.196	0.608	0.660	0.642
SVM	0.469	0.572	0.578	0.570	0.841	0.650	0.480	0.651	0.574
RF	0.473	0.495	0.498	0.498	0.764	0.501	0.495	0.471	0.498
Nb	0.320	0.495	0.542	0.565	0.839	0.595	0.495	0.588	0.533

**Table 10:** Comparison of proposed work with existing work.

Work	Purpose	Classifiers	Parameter count	Accuracy (%)	Sensitivity (%)
Pucer et. al.^36^	Arrhythmia beat detection	Discrete Morse theory	2	92.73	73.35
Raj et al.^74^	Arrhythmia detection	PS optimized LS twin SVM	3	99.11	91.47
Zhu et al.^75^	Arrhythmia detection	Maximum Margin clustering	2	95.9	97.4
		with immune evolution			
Donna et al.^76^	Heart disease	KNN	3	96.68	100
Ismail et al.^77^	Heart disease	SVM	4	79.71	NA
Luxmi et al.^12^	Heart disease	CFS+PSO+MLP+MLR+c4.5	4	88.4	NA
Our contribution diagnosis of cardiac health condition	Arrhythmia and abnormal beat	Proposed classifier	**6**	**99.97**	**99.98**
	Heart disease	SVM		**99.8**	**99.4**
		RF		**99.9**	**99.96**
		Naïve Bayes		**98.0**	**96.1**
		KNN		**99.4**	**98.0**
		NN		**99.8**	**99.4**
		SVM		**73.1**	**79.9**
		RF		**78.3**	**78.6**
		Naïve Bayes		**80.3**	**82.2**
		KNN		**69.0**	**72.0**
		NN		**80.6**	**80.4**

In the comparison, we analyze that in Table 6, both neural network and random forest achieved the highest performance in all metrics. However, the performance of SVM and KNN is relatively lower than the others. Furthermore, based on the results discussed in Table 6, we discovered that the neural network’s performance and naïve Bayes achieved the highest performance in all metrics. Secondly, the performance of random forest also achieved remarkable results in all metrics, whereas SVM and KNN performance have the lowest efficiency compared to the others.Consequently, based on the results obtained in Table 7, this study observed that the average performance of the Neural network using our DB-1 and DB-2 results is much higher than the rest of the classifiers. However, Random Forest and Naïve Bayes also achieved remarkable results, whereas the support vector machine and k-nearest neighbor performances present the lowest efficiency. Based on the results in Table 9, we observed that neural networks, naïve Bayes, and random forests exhibit less uncertainty compared to others. In contrast, the results for KNN and SVM are acceptable.Utilizing the information theory concept to assess the level of uncertainty in the classifier is motivated by the understanding that a significant level of uncertainty in the models is not suitable to implement in the Internet of medical applications.To underscore this contribution in our study, it is analyzed that we achieved the lowest level of uncertainty, less than 0.5, in sensitivity for all models. However, only KNN returns a slightly higher range of the level of uncertainty. These results in Table 9 demonstrate the suitability of implementing these models in real-world scenarios.

This represents an innovative contribution to our research, and we did not come across a similar study in the existing literature. As a result, we faced challenges in conducting a comparative analysis with state-of-the-art methods.

### The comparison of the proposed study with related work

In this section, the proposed method is compared with state-of-the-art methods. Our study conducts unique experiments to explore the information entropy of learning algorithms. No state-of-the-art methods related to our investigation currently exist. We utilize two natural datasets concurrently within a single framework to analyze cardiac health conditions. We could not find any study that incorporates both datasets in their investigations to predict cardiac health in terms of arrhythmia and heart disease.

To demonstrate the effectiveness of our suggested technique, we discussed recent advancements in the field of arrhythmia and heart disease detection. A comprehensive summary of the results is presented in Table 10 regarding accuracy (acc) and sensitivity (Se). The state-of-the-art method achieves highly accurate classification performance. However, implementing our proposed method enhances the analysis of ECG signals and heart disease using non-invasive clinical attributes. The results of our proposed classifiers achieved the highest performance using (DB-1), whereas the performance of our proposed classifiers is slightly lower using (DB-2). The reason behind the lower accuracy and sensitivity for heart disease detection was that the dataset required further preprocessing steps for better classification results. However, state-of-the-art studies only focus on heart disease classification using several methods.Based on our study, for the preprocessing of the DB-2 dataset, we focus only on the feature selection phase and outliers’ removal. Therefore, we observe that heart disease classification using UCI repository datasets requires high preprocessing methods to achieve overwhelming performance from learning algorithms.

### The limitations

After analyzing the results, it becomes clear that there is still potential for efficient preprocessing in (DB-2). Furthermore, our study delves into the analysis of ECG signals, concentrating on general arrhythmia and normal and abnormal beats. However, the remaining classes requires attention in detection for instance atrial fibrillation, ventricular fibrillation,cardiomyopathy.

## Conclusions and future work

A feature preprocessing approach is presented in this work to identify the cardiac health condition in terms of normal, abnormal(arrhythmic beat), and heart disease using two datasets. Furthermore, we introduce a new concept (information entropy) for determining classifier uncertainty levels when using medical datasets to overcome biased data diagnosis. This framework can assist researchers working in the fields of biotechnology, bioinformatics, and computational biology. Finally, the authors aim to conduct additional experiments based on the limitations discussed in section 5.3 in future work.

## Data Availability

The datasets generated and/or analysed during the current study are available in the Github repository, and the link is: https://github.com/q-mastoi/hmsystem.
